# Moving beyond the noise: geospatial modelling of urban sound environments in a sub-Saharan African city

**DOI:** 10.1038/s41598-025-06537-1

**Published:** 2025-07-01

**Authors:** Sierra N. Clark, Raphael E. Arku, Majid Ezzati, James Bennett, Ricky Nathvani, Abosede Sarah Alli, James Nimo, Josephine Bedford Moses, Solomon Baah, Allison Hughes, Samuel Agyei-Mensah, George Owusu, Mireille Toledano, Michael Brauer

**Affiliations:** 1 Department of Epidemiology and Biostatistics, School of Public Health, Imperial College London, London, UK; 2 School of Health & Medical Sciences, City St George’s, University of London, London, UK; 3 Department of Environmental Health Sciences, School of Public Health and Health Sciences, University of Massachusetts, Amherst, USA; 4 MRC Centre for Environment and Health, School of Public Health, Imperial College London, London, UK; 5 Regional Institute for Population Studies, University of Ghana, Accra, Ghana; 6 Department of Physics, University of Ghana, Accra, Ghana; 7 Department of Geography and Resource Development, University of Ghana, Accra, Ghana; 8 Institute of Statistical, Social & Economic Research, University of Ghana, Accra, Ghana; 9 Mohn Centre for Children’s Health and Wellbeing, School of Public Health, Imperial College London, London, UK; 10 School of Population and Public Health, The University of British Columbia, Vancouver, Canada

**Keywords:** Urban sounds, Audio, Machine learning, Noise, Ghana, Accra, Environmental public health, Environmental sciences, Risk factors

## Abstract

Cities encompass a mixture of artificial, human, animal, and nature-based sounds, which through long and short-term exposures, can impact on physical and mental health. Yet, most epidemiological research has focused on only transportation noise, leaving a significant gap in understanding the health impacts of other urban sound types, especially in sub-Saharan Africa (SSA). We conducted a large-scale measurement campaign in Accra, Ghana, collecting audio recordings and sound levels from 129 locations between April 2019-June 2020. We classified sound types with a neural network model and then used Random Forest land use regression to predict prevalences of different sound types citywide. We then developed a composite metric integrating sound levels with the prevalence of sound types. Road traffic sounds dominated the urban core, while human and animal sounds were prominent in high-density and peri-urban areas, respectively. Our high-resolution approach provides a comprehensive characterization of the complexity of urban sounds in a major SSA city, paving the way for new epidemiological studies on the health impacts of exposure to diverse sound sources in the future.

## Introduction

Cities have complex mixtures of mechanical, human, and nature-based sounds varying in space and time^1^ that can impact physical and mental health in different ways^2–5^. In many parts of the world, research, planning, and policy efforts have focussed on characterizing, regulating, and mitigating transportation noise, such as that from road, rail, and aircraft traffic^6–8^with the overwhelming majority of epidemiological evidence dedicated to these sources^6,9–12^. However, the reality is that in most cities, transport-noise coexists with sounds from other activities, such as construction and industrial works, neighbour noise, restaurants, bars, and night-life, shopping and commerce, and in some places, even religious activities^13–17^. While these other sources of environmental noise may also influence physical and mental health, they have not been well characterized in most cities. This is certainly the case in rapidly urbanizing cities around the world, such as in sub-Saharan Africa (SSA)^14,18–24^.

Nature-based sounds are generally perceived in city spaces as providing a calming and relaxing environment^13,25,26^. This is further supported by a limited number of studies that have demonstrated in controlled environments (i.e., virtual reality) that physiological stress recovery can occur after short-term exposure to nature-based animal (e.g., birdsong) or geophysical (e.g., water features) sounds^3,27–31^. Other types of sounds in cities can also evoke positive psychological responses, such as religious or culturally significant sounds (e.g., church bells)^13^ which have increasingly led to the inclusion of positive soundscape planning into urban design and policies^32–35^.

Exceeding global trends, cities in SSA are undergoing significant expansion and economic transformations, resulting in changes in urban land use (e.g., built-up versus natural areas)^36–40^ and transportation infrastructure^41–43^which will have an impact on the sound environment^32^. Fast growing SSA cities are now characterised by glaring urban transport problems, including traffic congestion, long commute times, and traffic related noise pollution^41,42,44,45^. Compared to European and North American cities^46–48^there is very limited research on the complexity of the sound environment in SSA cities, including on the health effects of exposure to environmental noise sources^45,49^. This poses a barrier to local urban planning efforts and noise mitigation efforts^32^but also for conducting epidemiological studies investigating the adverse, and even potentially beneficial, health effects of exposure to different sound environments, at city or population scale, in SSA.

Leveraging advancements in sensor networks and Machine Learning (ML) models^50,51^ we conducted a study that combined city-wide ML classified audio recordings and sound levels with spatial prediction modelling approaches (i.e., Random Forest Land Use Regression) to characterise and spatially predict the diverse sound environment in a major sub-Saharan African city over a period of approximately one year. We also developed a composite index which reflects both the loudness of sound in an area and the frequency (i.e., prevalence) of different sound types. The data can aid local urban sound planning and design efforts in Accra and support future planned epidemiological studies investigating the direct and interactive effects of exposure to different urban sounds. Lastly, our approach to characterising the complexity of the urban sound environment could be translatable to other SSA cities in the future to fill this major data gap and support urban sound policy design and planning in cities across the continent.

## Results

### Detected sounds from audio recordings

**Fig. 1:**
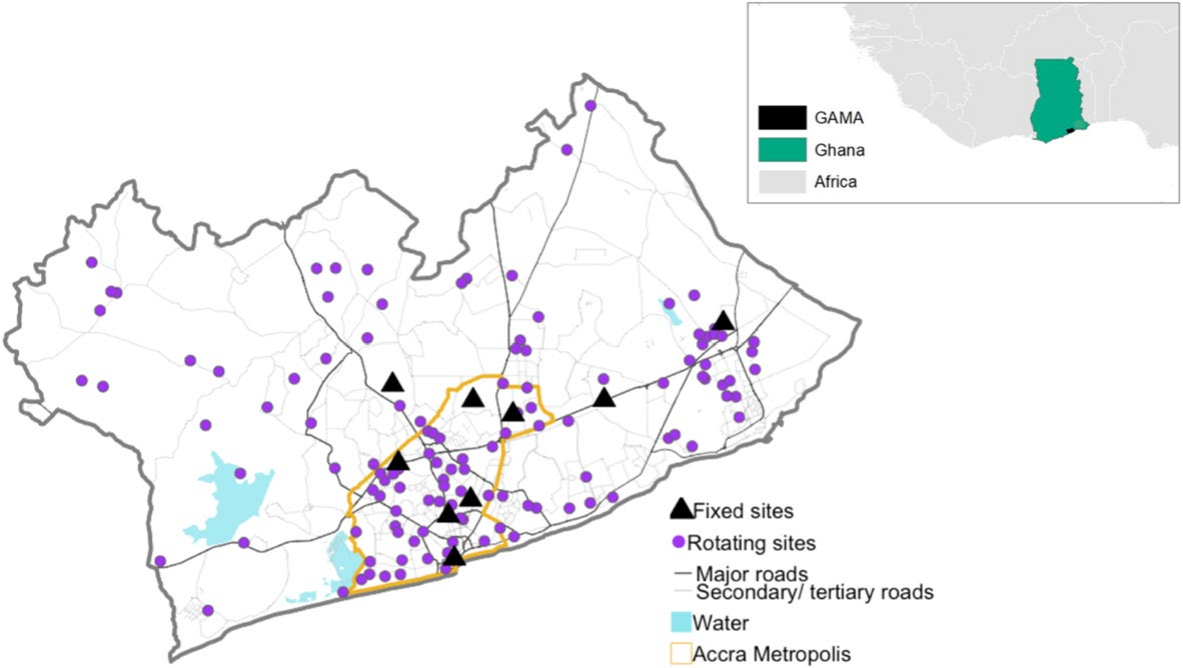
Locations of fixed and rotating measurements sites with environmental audio data in the Greater Accra Metropolitan Area, Ghana. Road network is from OpenStreetMap in 2019 ^52^ and the Greater Accra Metropolitan Area (GAMA) and Accra Metropolis boundaries are from Ghana Statistical Service (census year 2010)^53^. The inset shows background maps of Africa and Ghana (ESRI (Environmental Systems Research Institute)). A description of the land use categories used to sample the locations of the sites is in the Methods.

Valid audio recording data was obtained from 1,090 site-days spanning 129 measurement sites (Fig. 1) across the Greater Accra Metropolitan Area (GAMA, a 1500 km^2^ metropolis with ~ 4 million residents) which were passed through a pre-trained neural network to classify different types of city sounds (see Methods section for details and model accuracy metrics). At the measurement locations, road-transport sounds were the most prevalent in both the day and night-time (median percentage of time present in the day (78%) and night (58%), while animal and insect sounds were also common, particularly at night (median: 55% of time present). The median percentage of time that music sounds were present was between 5 and 6% and < 1% for geophysical nature sounds (see Supplementary Information (SI)).

### Spatial model predictive accuracy and variable importance

The amount of vegetation (Normalised Difference Vegetation Index (NDVI)) in an area was an important predictor of sound type prevalence in almost all Random Forest Land Use Regression (LUR) models. In addition, predictor variables representing road-traffic (e.g., road-networks) were important in both the road-transport and animal and insect sound models. Some of the most important variables in the music, speech, and nature sound models were related to population and building density and land use (more details in the SI).

The median absolute errors (MAEs) of the road-transport day and night models ranged from 10 to 16%, and the correlation of predicted and observed values ranged from 0.68 to 0.69, when holding out 10% of random measurement site data 10-times from the model building process (CV_10%sites_). The accuracy metrics for the animal models were similar (see SI). The MAEs of the speech models ranged from 4 to 13% with CV_10%sites_, and the correlation of predicted and observed values ranged from 0.53 to 0.60. The music LUR models were more accurate at predicting low levels of music sound prevalence than they were at predicting higher levels of music sound prevalence since there were limited data points at the higher ends of the distribution to train the model. This is reflected in the fact that the MAE was relatively low, being driven down by the majority of observations at the lower end of the distribution that were predicted correctly. The correlation between the observed and predicted values for music sounds was moderate-low (CV_10%sites_: 0.38–0.42). The geophysical nature sound LUR models could not generalize and predict accurately to new locations when the values were greater than 0% prevalence, as the distribution of nature sounds at our sites were highly skewed towards zero (see model performance statistics in SI). Thus, we do not present geophysical nature sound predictions further in this paper.

### Spatiotemporal variation in predicted sounds

**Fig. 2:**
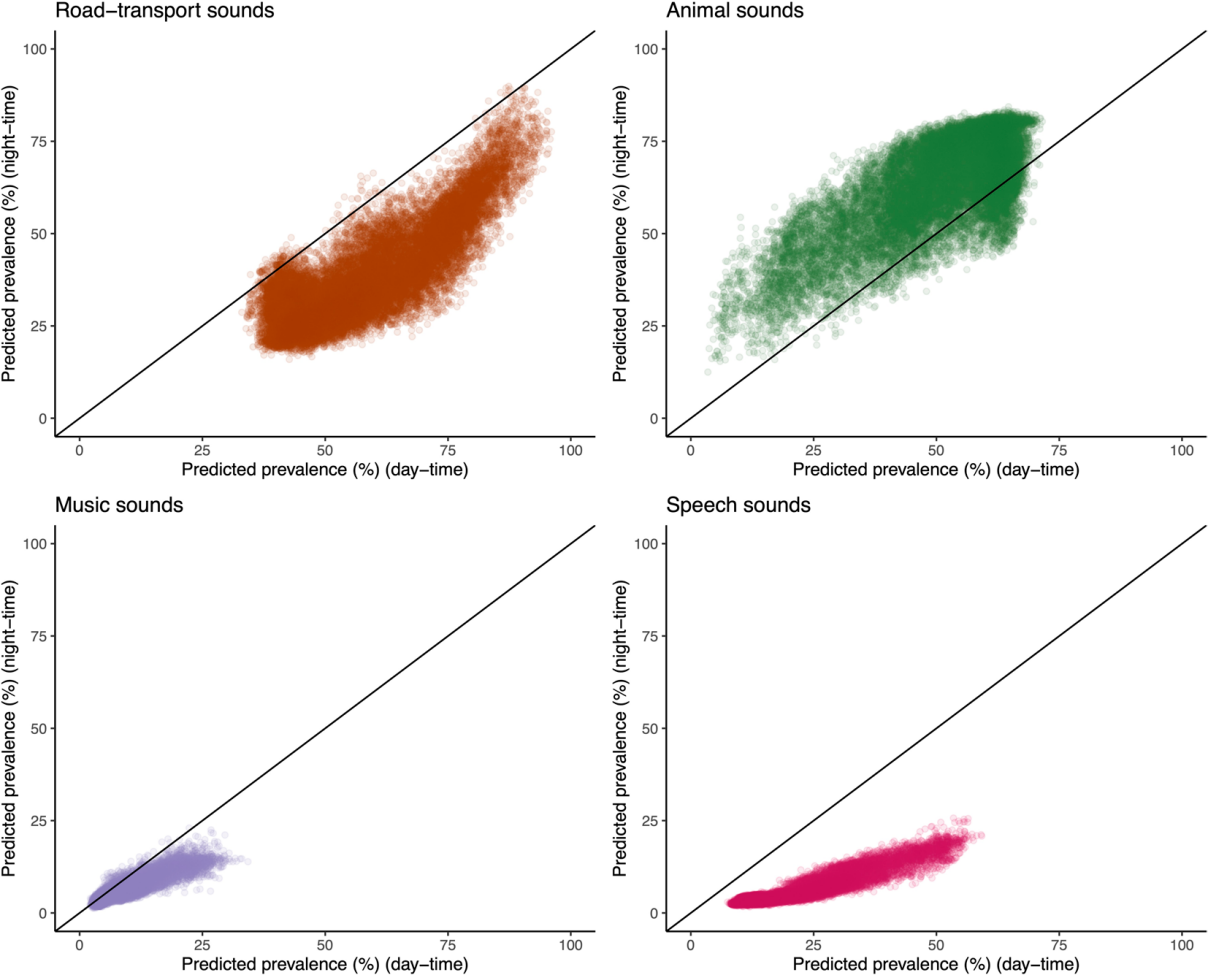
Correlation of day and night-time sound type predicted prevalence across Greater Accra Metropolitan Area (GAMA). The horizontal 45-degree black line indicates where a perfect correlation would have been.

**Fig. 3:**
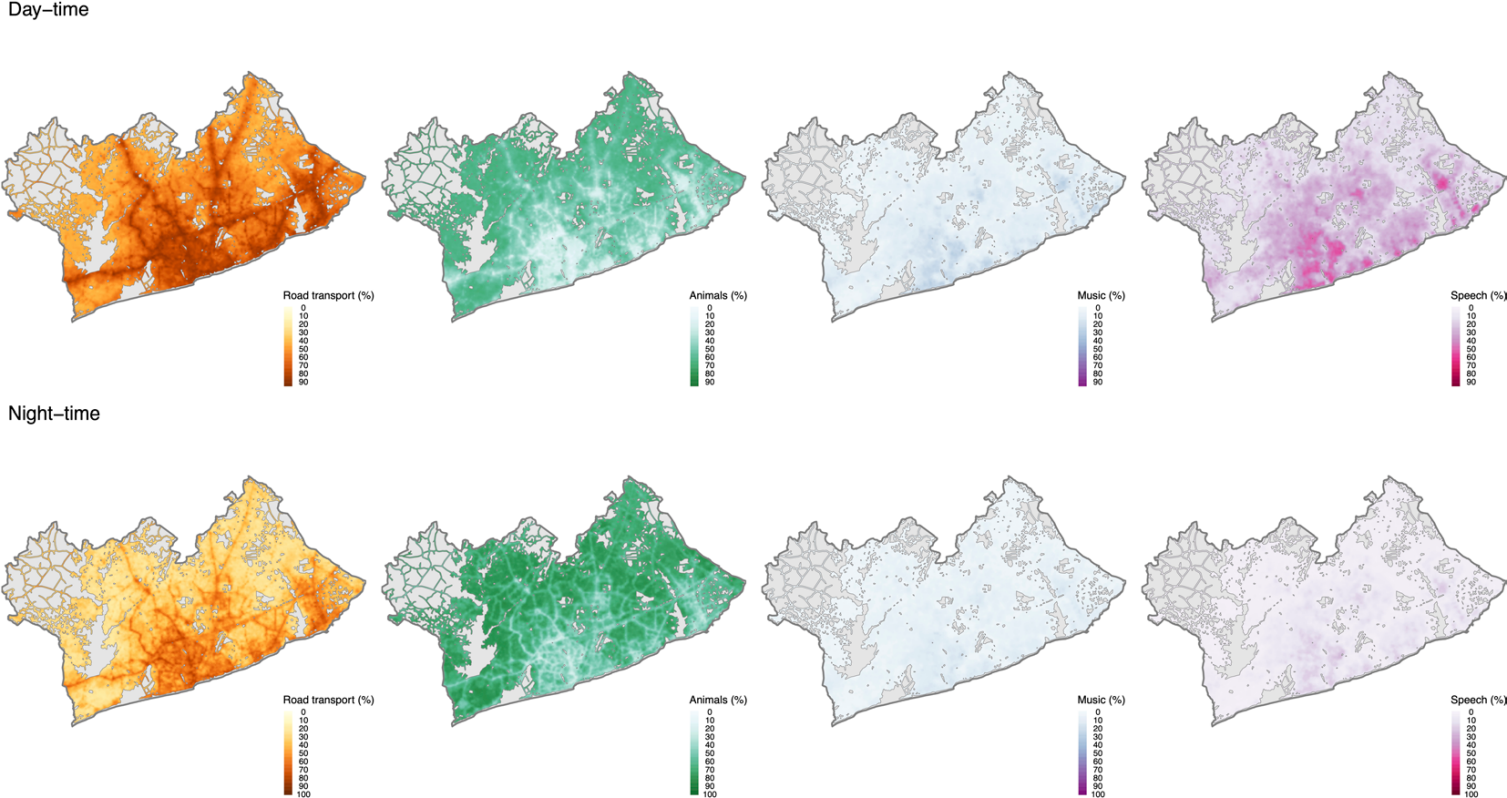
Predicted percentage of the day- and night-time that road-transport, animal and insect, music, and speech sounds were present (%) at locations in the Greater Accra Metropolitan Area (GAMA). Grey areas on the map represent areas excluded from prediction as they are considered to be out of sample (e.g., water bodies, forest/ grassland with no human settlement and no roads).

Predicted road-transport sounds dominated the soundscape of the GAMA, present nearly half of the time during the daytime (median [IQR]: 56% [46, 69]) and a significant portion of the night-time as well (median [IQR]: 35% [28, 46]) (Fig. 2). Though the prevalence of road-traffic sounds were generally lower at night-time (Fig. 2) and these sounds were far from spatially uniform, with notable variation across the region (Fig. 3). The quietest zones were the northern peri-urban residential areas, where daytime road-transport sound prevalence barely reached 30% and dropped below 10% at night. As expected, the highest concentrations were found near major road networks and in densely populated urban centres like Accra Metropolis (AMA), where road-transport sound prevalence peaked at 80% during the day and 62% at night. Eastern urban hubs like Tema and Ashaiman also experienced high levels, with road-traffic sounds present 69% of the day and 46% of the night. Interestingly, a few locations (< 2%) in the GAMA had more road-traffic noise at night than during the daytime.

The spatial distribution of predicted animal and insect sounds showed a strong inverse correlation with road-transport noise (Pearson correlation: -0.90 during the day, -0.87 at night). During the daytime, animal and insect sounds were typically present about a third of the time in Accra Metropolis (median: 31%) and near major roads (median: 35%). At night, the prevalence of these sounds increased, especially in the peri-urban areas of northern GAMA. Across the four sound types, animal and insect sounds were the only sound category where at almost all locations the percentage of time present was higher at night than during the day (Fig. 2). Music and speech exhibited similar spatial patterns (correlation: 0.84 at night to 0.91 during the day), with the highest levels observed in densely populated neighbourhood’s, particularly within Accra Metropolis. (Fig. 3). At all locations across the GAMA, speech sounds were more prevalent during the day-time than the night-time, as would be expected. A very small number of locations (< 10%) had a higher percentage of time where music sounds were present during the night-time, compared with the day-time (Fig. 2).

### Sound type index

**Fig. 4:**
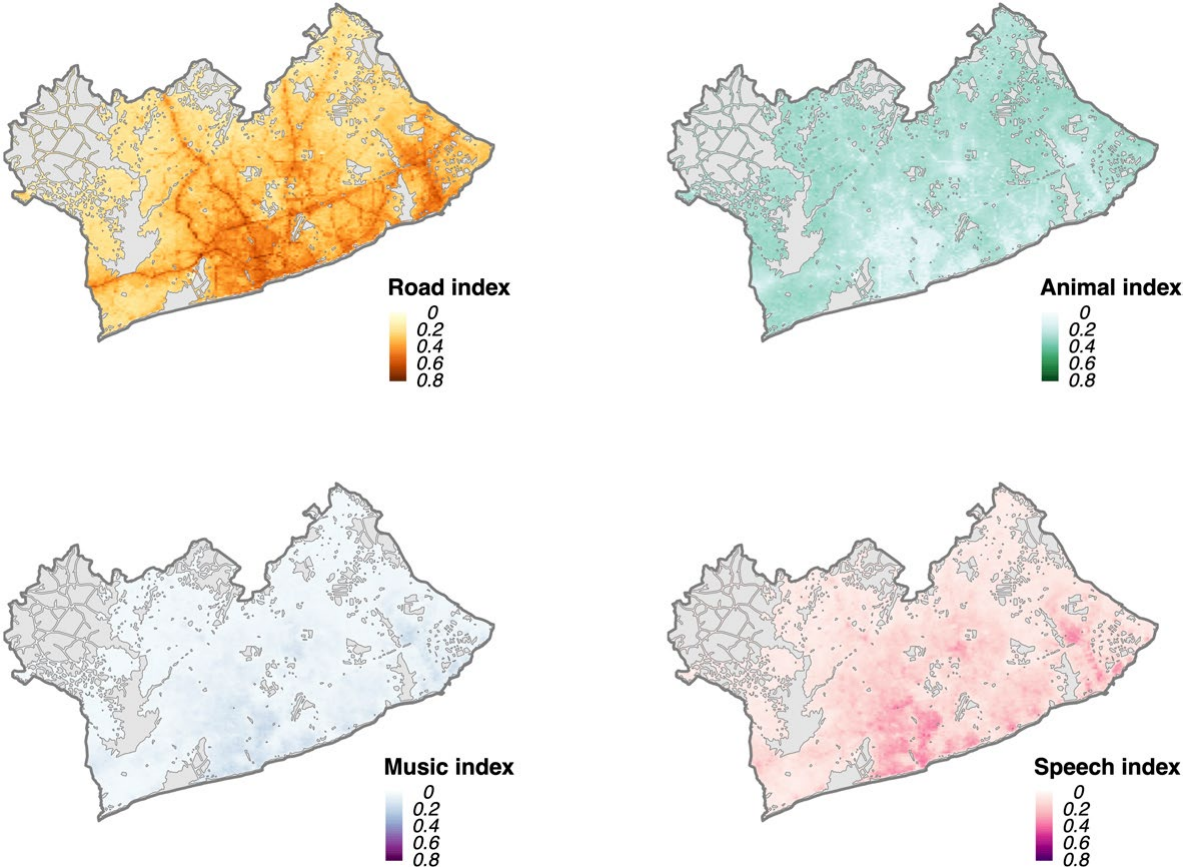
SoundType index predicted values for the day-time in the Greater Accra Metropolitan Area. Grey areas on the map represent areas excluded from prediction as they are considered to be out of sample (e.g., water bodies, forest/ grassland with no human settlement and no roads).

**Fig. 5:**
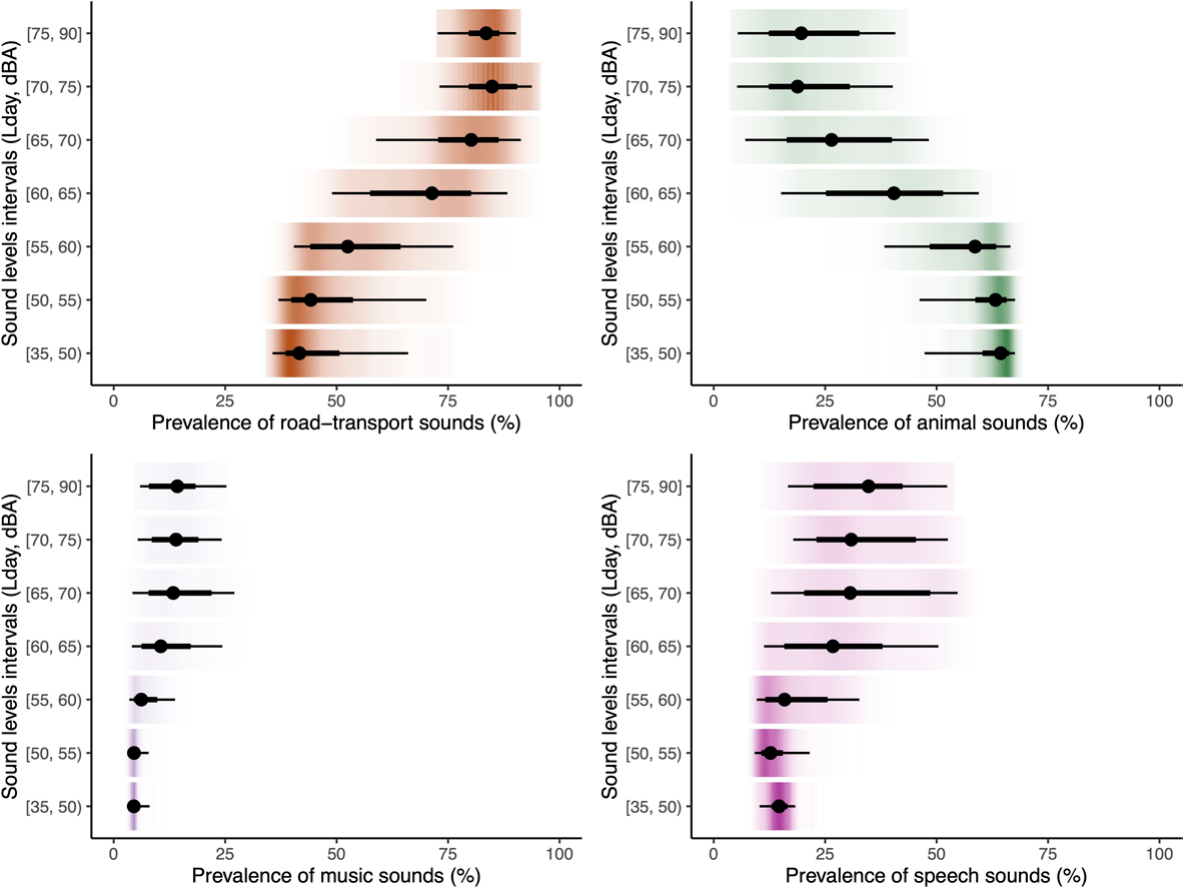
Distributions of daytime predicted sound type prevalence (% of time present) across areas with varying predicted sound levels (L_day_, dBA). Sound levels for each area in the GAMA were predicted from a LUR model described elsewhere^54^. Circular points represent the sample median, black horizontal lines the quartile range, and the coloured regions the density of the sample distribution. Night-time values are shown in the SI (Figure SI2).

For applications with city-scale measurements, we derived a composite metric (called the ‘SoundType Index’) which represents both the frequency of sound types (%) and measured sound levels (dBA) across space and time (see Methods for details). The road-transport soundtype index values had the greatest variation (see Table 1) but also the highest values, on average, compared to other sound types (Fig. 4), particularly in the Accra Metropolitan area reflecting both the fact that this type of sound is common, but also is present with higher measured sound levels (Fig. 5). In the night-time, the road-transport soundType index values were lower than day-time (Table 1). While animal and insect sounds were relatively prevalent in the GAMA, no areas had an animal and insect soundType index exceeding > 0.5, reflecting the fact that areas in the GAMA where there were a high prevalence of animal and insect sounds were also generally the places with lower sound levels (i.e., in the peri-urban periphery) (Fig. 5 and Figure SI2 in the Supplementary Information)). The median music soundType index values in the day and night were low (< 0.03, min: 0.0, max: 0.21), largely a result of the lower levels of predicted music sound prevalence in the GAMA compared with other sound types. Though the median music soundType index values were higher in Accra Metropolis, reflecting the increased prevalence of that sound type in the city-centre.

**Table 1:** Predicted sound type prevalence (average % of time present), predicted L_day_ and L_night_ sound levels (dBA)^54^and combined soundtype index values for locations in the greater Accra metropolitan area (GAMA) and the Accra metropolis (urban core). Data are summarized as median and interquartile ranges (IQR).

	GAMA		Accra Metropolis	
Sound level (dBA)	Day-time	Night-time	Day-time	Night-time
	58.4 (56.1, 60.7)	51.2 (49.6, 53.3)	62.3 (59.4, 64.9)	54.6 (52.0, 57.5)
Percentage of time sound types are present (%)	Day-time	Night-time	Day-time	Night-time
Road-transport sound prevalence	55.8 (46.1, 69.4)	34.7 (27.9, 46.3)	75.7 (67.8, 80.3)	55.1 (46.7, 63.8)
Animal and insect sound prevalence	55.2 (43.4, 62.2)	65.6 (55.4, 73.5)	31.3 (20.8, 44.8)	47.3 (38.1, 56.5)
Music sound prevalence	6.5 (4.7, 10.2)	5.1 (3.7, 6.9)	13.8 (8.7, 19.2)	9.5 (6.2, 12.3)
Speech sound prevalence	17.0 (12.8, 26.1)	4.2 (3.3, 6.7)	31.3 (23.3, 42.8)	9.6 (5.5, 14.3)
SoundType index (range: 0–1)	Day-time	Night-time	Day-time	Night-time
Road-transport sound index	0.27 (0.20, 0.35)	0.12 (0.09, 0.18)	0.41 (0.33, 0.47)	0.23 (0.18, 0.29)
Animal and insect sound index	0.25 (0.21, 0.27)	0.23 (0.20, 0.26)	0.17 (0.12, 0.22)	0.19 (0.16, 0.22)
Music sound index	0.03 (0.02, 0.05)	0.02 (0.01, 0.03)	0.08 (0.04, 0.11)	0.04 (0.02, 0.05)
Speech sound index	0.08 (0.05, 0.13)	0.02 (0.01, 0.02)	0.17 (0.11, 0.25)	0.04 (0.02, 0.06)

GAMA: Greater Accra Metropolitan Area.

### Discussion and conclusions

As we have shown, low-cost audio recording devices^55^ coupled with machine learning^56–59^ and spatial prediction models can help fill the data gap on sound environments and sources of noise in cities in SSA and elsewhere^60^. The detailed information that we collected and modelled on urban sounds, and their spatial and temporal patterns, can be of aid to local policy makers and city planners, to identify areas with acoustic qualities that could be mitigated (e.g., traffic noise pollution) or conversely areas where sounds might be preserved or enhanced (e.g., constructing parks which enhance birdsong). Exposure scientists and environmental epidemiologists can also leverage the approaches presented in this paper to generate city-scale, high-resolution data on the prevalence of different types of sounds in cities, allowing for the inclusion of these exposure surfaces in epidemiological studies investigating the adverse, as well as potentially beneficial, health effects of exposure to different types of sounds along with the more traditional metrics representing sound levels and events from transportation sources (i.e., L_den,_ L_night_).

Road-transport and animal sounds (e.g., often representing bird song) were regularly patterned across the city and the models were able to predict gradients of prevalence with a good degree of accuracy. Unfortunately, the Random Forest LUR models were not able to accurately predict geophysical nature sounds due to the lack of representation of the model training data at prevalences higher than 0%. In the future, additional audio measurements in targeted nature-based spaces in Accra could be collected, classified, and included in the models training dataset to improve the spatial prediction performance. Furthermore, many types of geophysical nature-sounds, such as the timing of rainfall events or the rustling of leaves during windier days, are not patterned across space but are patterned across time. Therefore, it is no surprise that our spatial predictors were not able to capture the variation in the geophysical nature sound category across the city in our models, which may be more temporal in nature.

We found that during the day-time, spatially predicted road-transport, music, and human speech sound prevalences were generally higher than during the nighttime. However, animal and insect sound prevalences were generally higher at most locations during the night-time than in the day-time (Fig. 2). With five sound type categories (including attempting to model nature-based geophysical sounds) and two time periods (day and night), this resulted in the development of 10 separate models, representing diverse predicted trends over space and time. However, we recommend that future work be carried out to further refine and develop models with an hourly resolution (as we previously developed for sound level metrics in Accra^54^) and additionally to capture and define the evening period in a distinct way. The evening period represents a key time window in which individuals are likely exposed to distinct sound profiles while they are at home, yet not yet asleep, and also captures the period in which the evening traffic commute begins to subside in some locations. It would also allow for the development of a 24-hour based metric, similar to L_den_, which is commonly used to estimate health effects of exposure to noise across the full 24-hour day and penalises different time periods (night-time, and evening) based on increased sensitivity to noise during these periods. Further methodological work would need to be carried out to consider how to develop such a metric with these sound type data inputs, and in particular how to handle penalties for different types of sounds.

We found that in the GAMA, predicted road-transportation sounds were pervasive and generally accompanied with higher sound levels. Demand for motorized transport has rapidly increased in Accra over the last few decades as the population^61^ and the city limits have continued to expand^61^. This has resulted in worsening traffic congestion, particularly in Accra Metropolis^42,61^. Private vehicles or privately owned minibuses/vans known locally as tro tros are currently the primary means of getting around the city’s main arteries. Greater Accra also overwhelmingly has the highest proportion of registered vehicles in Ghana, more than half of the total national number^42^. At the same time, urban growth has resulted in the re-development of natural lands, effectively decreasing the percentage of forested areas in the GAMA from 34% of land area in 1992 to 6.5% in 2015 ^39^, particularly in and around Accra Metropolis. However, many neighbourhood’s in Accra have dense and mixed-land use, resulting in a mixture of different types of sounds contributing to the overall sound environment, which was reflected in our findings. So, while measurements were taken along the road-side, road-traffic sounds were not the only contributors to the measured L_day_ and L_night_ levels (dBA). Many roads in Accra and the accompanying sidewalks have mixed uses and functions, such as road-side commercial sales, outdoor dining/events, and even play for children^62^. As we have shown, the collection of audio recordings in noise measurement campaigns can provide additional contextual information on the sources of the measured sound levels, which do not automatically distinguish the source themselves (i.e., without detailed frequency analysis).

Urban green spaces (with trees/plants) or private gardens can facilitate what are typically described in soundscape studies as ‘tranquil/desirable’ sounds^13^ by providing a habitat for birds as well as being a source of sound itself (e.g., rustling leaves). Certain types of vegetation may also reduce noise from other sources in the surrounding environment by acting as a natural attenuator through absorption and diffusion^63,64^. While controlled experimental or neighbourhood-scale soundscape studies have found that geophysical nature-based and animal sounds such as birdsong have been linked to enhanced feelings of subjective wellbeing and physical markers of stress reduction^3,4,29,65,66^these relationships have not been confirmed in population-based large-scale epidemiological studies. The SoundType index, which jointly represents both the sound level and the frequency of different sound types in an area, derived from measurements and LUR models, is well placed for inclusion and testing in future epidemiological studies in Accra of both the negative, as well as potential beneficial, health impacts of exposure to different types of sounds. For example, the derived exposure datasets will be linked up with new health data in Accra, collected as part of the Accra Schools Health and Environment study (ASHES), a cross-sectional study of over 900 school children’s health and development, as well as the Accra Birth Cohort (ABC) study, longitudinally tracking birth outcomes and developmental health impacts among pregnant women.

### Strengths

The data collection campaign in Accra produced millions of sound level and audio records covering every hour of the day, day of the week, and month of the year at diverse locations in the city. Our systematic selection of monitoring equipment that was cost-effective, energy-efficient and battery powered, lightweight, and socially unobtrusive optimized data collection and was less labour intensive in a setting with unreliable electricity. Modelling and spatially predicting sound type prevalences in high-resolution at city scale using audio recording classifications and LUR models, to our knowledge, is one of the first few attempts to do so in any urban setting. The use of Random Forest modelling allowed for computationally efficient and relatively accurate prediction of many of the sound types. While there is area for improvement (as discussed below), this work shows potential applications for other cities in SSA and elsewhere.

### Limitations

There are several limitations to this work. While we used a wide variety of spatial predictor variables, we were not able to obtain spatially and/or temporally resolved information on traffic flow, volume, and fleet composition. Inclusion of this information could have improved model predictive accuracy, particularly for transport-related sounds, as we have already shown in previous work with measured sound levels (non-source resolved)^67^. We had to make the assumption that the spatial predictors were stationary in time and represented the period when the audio recordings were taken. This assumption may not be true for all spatial predictors as the census data which was used to estimate population density was generated in 2010 and the dataset used to estimate land cover dates to 2014. The temporal misalignment of some of the predictor variables may be especially relevant for the urbanizing periphery areas of the GAMA.

The different types of sounds identified from each audio recording by the sound classification neural network (as described in Clark et al. 2021 ^45^) is partly a function of how *clear* the recording was, how *loud/prominent* the specific sound was in the recording, but also the neural network itself and the unique nature of the training dataset. It is also likely the case that sometimes specific sound categories were not identified within an audio recording because they were masked by other sounds (e.g., road-traffic masking out sounds of rustling leaves), which could be a function of the fact that the audio recorders are low-cost, and inevitably lower precision and accuracy, instruments. However, we do not believe that masking effects had a major impact on the spatial distribution of the predicted sound type prevalence’s for the following reasons: the accuracy of the neural network (based on human labels) was fairly good - between 76 and 93% for road-traffic, animals and insects, human speech, and music – and the relatively small percentage of non-accurate labels could be from either masking or misclassification^45^; also the spatial patterning of sound sources that were identified visually from analysis of time-lapse camera imagery collected at the same locations^68^ largely followed the same patterning found with audio analysis. Finally, we were interested in those sounds which were clearly identifiable in the audio recordings, and supposedly by the human ear also, which better reflects the human experience of the sound environment at each place and time. Also, due to data limitations, we had to aggregate classifications into broad categories, within which there may be different human perceptions and preferences for different sound types (e.g., preference some bird songs and calls but not from other birds). As the acoustic classification model returned the top 3 predicted sound classes, we were able to detect overlapping sounds in each place and time. However, the model is not able to provide an estimate of how predominate each sound type is, and therefore they are counted equally, which is a limitation of this work. To manage storage constraints and maintain privacy, we recorded 10-second audio clips every 10 min—yielding six samples per hour across all hours and days. We assume this temporal sampling strategy sufficiently captured the variation in sound sources over time. Still, implementing this type of urban acoustic monitoring may not be practical in all contexts, especially where different legal and privacy frameworks apply. Some alternative approaches rely on high-resolution (L125ms) spectral data, though these require more advanced—and often more costly—equipment, which can limit data collection capacity. Lastly, while our sound classification accuracy could likely be improved by either building a custom model or retraining an existing one using local audio data, these steps were beyond the scope of the current project. Furthermore, while our underlying acoustic classification models used *Audio Set*, other open-access datasets used to train urban noise models exist, such as *UrbanSound8K* or *ESC 50*. Future work should investigate the applicability of these datasets to African urban contexts.

## Conclusion

In conclusion, we conducted a study that integrated city-wide audio recordings and sound level measurements with machine learning techniques to characterize and spatially predict the varied sound environment in a major sub-Saharan African city. Additionally, we developed a composite metric that reflects both the magnitude of sound levels in specific areas and the prevalence of different sound types, showing a new approach for how the complexity of urban sound environments can be captured and characterised through measurements. The insights gained from this research can assist in local urban sound planning and design initiatives in Accra, while also supporting future epidemiological studies investigating the direct and combined health effects of exposure to diverse urban sounds on city residents, filling a major evidence gap in SSA. Moreover, our distinctive approach to characterising the complexity of urban sound profiles holds potential for application in other SSA cities, addressing a significant data gap and supporting the development of urban sound policies across the continent.

## Methods

### Study area

Our study was conducted in the Greater Accra Metropolitan Area (GAMA, 1500 km^2^), the most densely populated area in Ghana and the political, economic and administrative capital. This metropolitan region (~ 4 million people in 2019) includes Accra Metropolis as its core (~ 2 million people), Tema to the east (~ 200,000 people)^69^and the expanding suburban municipalities to the northeast and northwest.

### Data collection

#### Environmental monitoring campaign

Study design and data collection procedures are described in detail elsewhere^70^. Briefly, we conducted environmental monitoring at 146 locations in the GAMA for 7-day (136 rotating site locations) and around 1-year periods (10 fixed site locations) between April 10th 2019 and June 11th 2020 (Fig. 1). The measurement campaign was briefly interrupted in a period between March and May 2020 due to COVID-19 pandemic lockdown. The fixed sites were purposefully chosen through substantial knowledge of the local area to represent a diversity of land use, socioeconomic, and transport features. The rotating sites were selected through stratified random sampling with strata representing land use^71^ and sites were oversampled in Accra Metropolis which is the most populous area (~ half of the population in GAMA lives in Accra Metropolis). The four land use categories from a 2014 World Bank map included: medium/low-density residential; high-density residential; commercial, business and industrial areas; and ‘other’ areas (e.g., parks, forest, agricultural areas) (details in Clark et al. 2020 ^70^). Rotating sites were evening sampled across these four categories and 50% of sites were additionally located within Accra Metropolis. Briefly, medium/low-density residential are typically formal residential areas and can have paved or unpaved roads which are double or single lane. The housing property can have yards, fences, walls, and/ or driveways and there is a clear demarcation of where one home ends and another begins. High-density residential are typically informal residential areas and could be classified as shantytowns or slums. Roads (paved or unpaved) are typically narrow, sometimes unidentifiable, and buildings are typically small with a lack of a clear demarcation of where one home ends and another begins. Other areas are the least built-up areas and can have an abundance of forest land, grass land, shrubs, barren land, water, and/or be classified as a park. Commercial, business and industrial (CBI) areas are places which are either dominated by commercial, business, industrial, or government activities. These areas can have large buildings, though that is not a requirement.

As we previously reported in Clark et al. 2021 ^45^, we also overlaid these site locations onto city-wide geographic data representing vegetation levels and population density and summarized the levels of these descriptors by our land use categories. Specifically, population density (people/km^2^) within census enumeration areas (Median EA area: 0.03km^2^) was obtained from the 2010 Ghana National Population Housing census. This is the most up to date and reliable record of population distribution within Accra at the small-area level. We obtained estimates of vegetation by calculating the Normalized Difference Vegetation Index (NDVI) from 30 m spatial resolution Landsat 8 satellite imagery collected at the mid-point of the campaign in January 2020. This imagery also had the lowest amount of cloud cover in the potential set^72^. NDVI values close to zero indicate no green vegetation (mostly built-up areas), and values close to + 1 indicate a high abundance of available vegetation. The mean NDVI level was 0.09 in high-density residential areas, 0.13 in medium/low-density residential areas, 0.10 in CBI areas and 0.22 in ‘other’ peri-urban areas. Population density across the sites also followed a predictable pattern with the highest mean population density in high-density residential areas (35,833) and the lowest in ‘other’ peri-urban areas (1701).

#### Audio data collection and sound type classification

We deployed low-cost full spectrum, audio recorders (AudioMoth, Open Acoustic Devices (Oxford, UK)) with analog MEMS microphones to record the outdoor acoustic environment^45,70^. The AudioMoths were set to a sampling rate of 32 kHz to capture the majority of sound in the audible range^57^. The audio recorders recorded for 10 s every 10 min, recording onto a microSD card. Specifically, we collected audio throughout the day (6 samples per hour, every hour) and across days (Monday - Sunday) at each measurement site in the city. For the fixed sites, we additionally have data spanning 12 months of the year. At each measurement site, the monitors were housed in a functional and protective enclosure (see study protocol for additional information^70^) that was attached to a pole at ~ 4 (± 1) meters above ground and oriented towards the public streetscape. Audio recorders were programmed to run continuously for 7-day periods, after which they were either swapped out and installed at a new set of rotating sites or replaced with a fresh batch at existing fixed sites. Audio recorders were deployed regardless of weather conditions.

We also collected 1-minute integrated A-weighted sound levels (decibels, dBA) with Sound Level Meters that were co-located with audio recorders at each measurement site. We used the Noise Sentry Sound Level Meter (SLM) datalogger (NSRT_mk3) from Convergence Instruments, Canada to collect 1-minute integrated A-weighted sound levels. The device is small and rugged, built to withstand temperatures in the range of − 20 °C to 60 °C, protected against water and dust, and has a digital MEMS microphone. Prior to each monitoring session, the sound level meters (SLM) were calibrated with a CA114 sound calibrator at 94.0 dB ± 0.3 dB and 1000 Hz ± 0.5%. The Noise Sentry’s have a noise floor of 30 dBA. Pre-campaign sound level monitor-monitor precision tests showed good agreement (see Clark et al. 2020 ^70^) and the duplicate measurements that we conducted throughout the campaign at 16% of rotating sites indicated that the monitors did not drift from each other over time^45^. More details on the acoustic instruments, quality assurance and control checks can be found in the previously published study protocol^70^ and descriptive paper summarising the measurements^45^. We also previously developed the spatial LUR model for the environmental sound level metrics (L_day_, L_night_) and thus the development of these models is reported elsewhere^54^.

We discontinued data collection at one fixed site located near the ocean as initial recorders were damaged due to rust from the contact of the airborne sea-salt with the exposed circuit board. Furthermore, audio recordings from 16 rotating sites were lost due to equipment malfunction or theft. The final analysis utilized audio data from 9 fixed (yearlong) sites and 120 rotating (weeklong) sites (Fig. 1), consisting of a total of 1,232,640 site-seconds and 1,370,880 site-seconds of audio data from rotating and fixed sites, respectively.

In order to detect the presence or absence of different types of sounds across the measurement’s sites and over time, each 10-second audio recording clip was run through a pre-trained acoustic classification neural network^59,73^. Details on our use of the acoustic classification model (DEEP-Hybrid DataCloud project^73^ based on Changsong et al. 2018 ^59^) and its validation can be found in our previous paper^45^. In brief, the open-source model came pre-trained on the *Audio Set* dataset published from Google. *Audio Set* consists of 5,800 h of 10-second manually labelled YouTube videos and a structured hierarchical ontology of hundreds of sound classes. *Audio Set* is *weakly labelled* meaning that only the presence or absence of audio events are known in a clip. The neural network used in our analysis is based on the Multi-level Attention Model framework outlined by Changsong et al. 2018 ^59^. It processes each audio clip by first converting it into a spectrogram, which is then passed through a series of fully connected layers. Both the final and intermediate outputs of these layers feed into multiple attention modules. These modules are designed to focus on the most informative parts of the audio file while filtering out irrelevant elements like background noise. The outputs from these attention components are then combined to produce the final classification result.

After applying our own audio recordings to the pre-trained model, we obtained the model’s top three predicted sound classes (top 3 chosen as highest levels of accuracy) as the presence of different types of sounds in cities can be overlapping (i.e., not mutually exclusive). Because these are predicted from the model, we call these ‘detected’ sound classes. We then grouped the sound classes into higher order sound type categories for acoustic environment characterization that were adapted from the ISO 2018 standard for acoustic environment characterization^74^:

- road-transport sounds (e.g., engine sounds from motorized vehicles, honking),
- animal and insect sounds (e.g., birdsong),
- outdoor music (e.g., music played on the radio, people singing),
- human speech (e.g., people talking, laughing),
- airplane sounds,
- geophysical nature sounds (e.g., wind, rain, thunder).

We also had a category for ‘other’ which consisted of all of the remaining miscellaneous sounds. As we detailed previously^45^we tested the accuracy of the model’s predictions within each sound type category by comparing agreement against manual sound labels applied by a researcher that was blinded to the model results from 150 randomly selected audio files (1/3 from rotating sites, 2/3 from fixed sites). The randomly selected audio files from the fixed sites were representative across different times of the day. The accuracy of model predictions was 93% for music sounds (Positive Predictive Value (PPV): 71%; Negative Predictive Value (NPV): 97%), and 80% for speech (PPV: 83%; NPV: 79%), 79% for animal and insects sounds (PPV: 97%; NPV: 74%), and 76% for road-transport sounds (PPV 76%; NPV: 78%). Geophysical nature sound classifications had accuracy of 87% (PPV: 100%; NPV: 83%), though the frequency at which this sound type was present in the audio files was low (4 audio clips out of 150)^45^. Airplane sounds had an accuracy value of 82%, though the positive-predictive value was low, only at 4% (NPV: 99%). As such, we do not consider airplane sounds further in this study (more details published elsewhere^45^). We also did not model ‘other’ sounds in this study, as we assumed that it would be uninformative given that these are a mixture of miscellaneous sounds (e.g., ‘clatter’, ‘alarm, ‘smash’) with minimal spatial variation and predictable patterning in the prevalence across land use factors^45^.

To prepare the audio classifications for the city-wide models, we calculated the prevalence of the detected sound types at each monitoring location for each date of monitoring. We additionally separated the data by the day (06:00–21:59) and night-time (22:00–05:59) periods in line with Ghana Environmental Protection Agency (EPA) classifications. Prevalence was defined as the percentage of clips where a particular sound type was present (i.e., prevalence) at any given location and date, calculated as the proportion of that sound type predicted as present (yes = 1, no = 0) at that location and during the monitoring period over the total number of audio recordings collected at that location and during the monitoring period, multiplied by 100. These prevalence figures for each sound type category formed the dependent variables in our LUR model as described below.

#### Spatial predictor variables

To capture land use/ land cover, we used a raster dataset at 20 m resolution that mapped four land cover classes across the GAMA from Spot 5 imagery attributed to the year 2014. The land cover classes were (i) formal residential neighbourhoods with small regular planned buildings, (ii) informal residential neighbourhoods (e.g., shantytowns, slums) with small irregular buildings, (iii) industrial and business areas, with large buildings and, and (iv) other areas (e.g., parks, forests, water, grassland)^71^. To characterize vegetation in Accra, we calculated the Normalized Difference Vegetation Index (NDVI) from 30 m resolution satellite imagery^72^. We obtained a Landsat 8 satellite product held on the U.S. Geological Survey department website attributed to a cloud free day in January 2020. January was also considered as a mid-point in the measurement campaign. The Normalized Difference Vegetation Index (NDVI) was calculated from raster bands 4 and 5. We also retrieved data on waterways in Accra from OSM in 2019. To estimate building density, we made use of a spatial dataset of building footprints attributed to the year 2019/2020^75^. We estimated human population density from the most recently available Ghana census (2010) summarized within census enumeration areas (EAs)^53^. Census EAs are small geographic units with average population of 750–800 people and area 0.03–0.04 km^2^ within the GAMA. To capture road-traffic sources of sound, we used a road-network shapefile from OpenStreetMap (OSM) downloaded in 2019 ^76^. Road-types were grouped as (i) major roads (includes highways, motorways, trunk roads), (ii) secondary and tertiary roads (iii) minor roads, and (iv) all roads. To capture locations of human activity, we obtained the latitude and longitude locations of churches, mosques, hospitals, primary and secondary schools, restaurants, shopping centres and markets, bars, pubs, and nightclubs from the Google Places API in 2019. We also obtained locations of bus stations/terminals from Google Places as an indicator of both human activity and road-transport sources. Finally, we retrieved data on elevation above sea level from a digital elevation model (DEM) for Africa at 90 m resolution^77^. More detailed information on our predictor variables can be found in our environmental noise level land use regression (LUR) modelling paper^54^.

Consistent with our noise level LUR modelling approach^54^we created multiple circular buffers (50, 100, 200, and 500 m) around the locations of the measurement sites^78–82^. We then calculated the total length of each road category, the total number of bus stations/terminals, the total number of places of human activities, mean land cover, the average NDVI, the average population density, the total number of buildings, and the total length of waterways, within each buffer. We additionally calculated the distance of each measurement site to the nearest major road^83^.

#### Spatial modelling and prediction of sound types (random forest land use regression (LUR))

We used a land use regression (LUR) approach to model and predict the prevalence of different sound types across the GAMA. LUR models are commonly used to estimate spatial variability in air pollution within cities^84^ and have been increasingly applied to noise in high and middle-income countries^82,85–88^ and also recently in Accra^54^. An LUR model regresses spatially, and sometimes temporally, structured environmental data (e.g., sound source prevalence) on geospatial predictors that represent a range of factors in the urban environment that are associated with the emission, propagation and attenuation of sound. We specifically used Random Forest (RF) models for sound source prevalence prediction^89^. Random Forest are an ensemble learning method, made up of combinations of classification/regression decision trees, which on their own (single trees), are highly sensitive to being overfit. Though Random Forests overcome this drawback by combining multiple trees, which are made up of bootstrapped observations and a random subset of predictor variables^89^. Random Forests are also robust to the inclusion of many variables with little or no importance and place no distributional assumptions on the outcome variables^89^. Random Forest models have also been shown to achieve higher accuracy in predicting noise pollution related outcomes compared with linear regression in five Canadian cities^85^.

We constructed 10 different models. Five models for each sound type and separate models for the day and night-times. The dependent variables represented the average prevalence (i.e., % of time present) of the different types of sounds detected in the day (06:00–21:59) and night-times (22:00–05:59) at each monitoring location. Additionally, since we had an imbalance between the number of days of audio measurements at fixed sites (range: 227–322 days) compared with rotating sites (7-days), we randomly sampled 30 days of data from each fixed site to be included in the models. In this way, we took advantage of the additional temporal information captured at fixed sites across a 12-month period, without flooding the model with data collected from only a handful of locations (a similar approach was used to analyse objects detected from street view imagery collected in Accra from the same campaign^68^).

We selected the best buffer radii for each spatial predictor variable and corresponding model with the random permutation’s method. This method provides a robust measure of the importance of predictors in a Random Forest model^90^ and has been used by previous spatial noise modelling studies using Random Forest^85^. Specifically, this method generates a random permutation of a variable and calculates the difference in model predictive accuracy when the random permutation is made compared with when the variable’s true values are used. If a predictor variable is important, random permutations will result in a larger decrease in model accuracy compared to permutations of other variables. Finally, if we a priori considered a spatial predictor variable not to be sound producing in either the day or night-time, the variable was not included in that corresponding model. For example, we included the variable representing the number of *schools* in the daytime but not the night-time models, as we assumed that activities in and around schools produce sounds primarily during the daytime.

We fit Random Forest models with 500 trees each to the sound type dependent variables and a suite of spatial predictor variables. We evaluated the fit and external predictive accuracy of the final models with cross validation. We ran 10-fold cross validation holding out data from 10% of random measurement sites (CV_10%sites_). From the cross-validations, we calculated the accuracy of the model’s predictions by computing the median absolute error and mean absolute error, which are measures of deviation, and the mean error, which is a measure of bias. The importance of each predictor variable in each model was assessed with the same method used to choose the best buffer size, the random permutation method. We inspected the distribution of the model’s residuals over multiple time-intervals, including days of the week and months of the year. To check for spatial autocorrelation in the models’ errors, we (i) visually inspected maps of model ME across measurement sites and ran Moran’s I tests of spatial autocorrelation on the models’ residuals. We also combined the residuals across models to see if there was any clustering of model prediction error present in a combined way. Specifically, we first took the absolute value of the residuals from all models and then standardized them to have the same range, between 0 and 1. We then summed the standardized absolute residuals across the sound type models, for the day and night separately.

We generated predicted surfaces for the prevalence (% of time present) of different sound types for the day and night-time, for all areas in the GAMA, at 50 m resolution. Though we did restrict predictions to areas that our measurement sites were representative of so that we did not predict out of sample. Thus, we excluded areas that did not contain roads, were fully covered by water bodies, and/ or areas that were fully grassland/forest. In addition to mapping the predicted presence of sound types across the GAMA, we also summarized the median, interquartile range (IQR), minimum and maximum values within the GAMA and across spatial sub-groups such as within Accra Metropolis and by proximity to major, secondary/tertiary, and minor roads.

### Composite sound type index

Finally, we aimed to derive a composite metric which can characterize the multifaceted nature of measured sound in cities, by combining data on measured sound levels with the frequency of sound types across cities. To construct this composite metric (which we call the ‘SoundType Index’) we used the predicted surfaces of the prevalence of sound types (described above) combined with predicted surfaces of day-time (L_day_ (dBA)) and night-time (L_night_ (dBA)) annual average A-weighted equivalent continuous sound levels which were generated from LUR models described in a previous publication^54^. We first pre-processed the data so that both variables were on the same scale^91^. Specifically, we transformed the sound type prevalences from percentages (0-100%) to proportions (0–1). We also normalized the sound level data into the same range by subtracting each value by the data minimum and dividing by the data range. While this allows for comparison of levels within our data and across locations within Accra, it may hinder comparison of this composite metric when applied to other cities and regions around the world where the variation and range of sound levels can differ to that found in Accra.

**Fig. 6:**
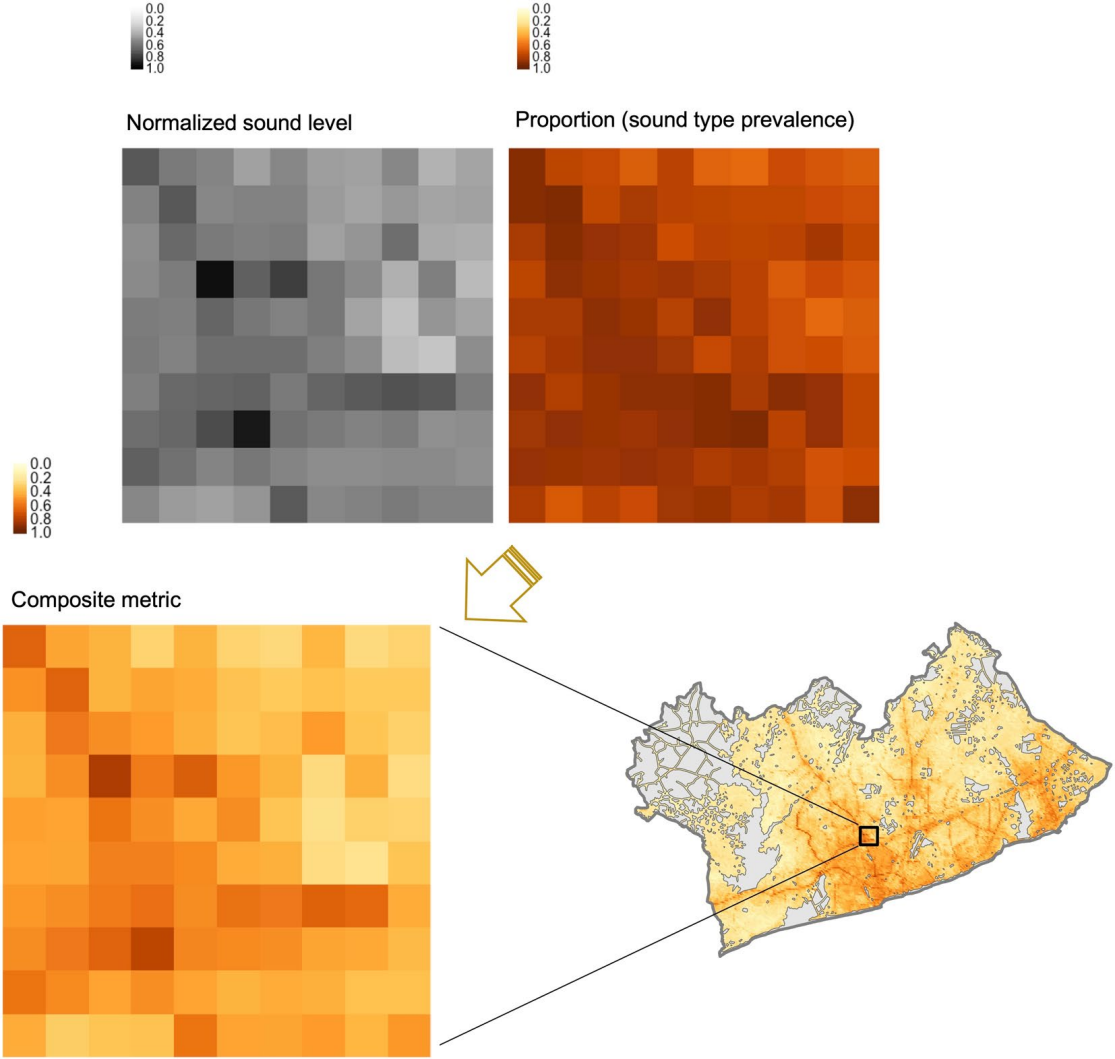
Graphical example of the creation of the SoundType Index. Example shown represents the day-time road-transport index. The figure shows the geometric aggregation of normalized day-time sound levels (Normalized (L_day_)) with the day-time proportion of time that road-transport sounds were predicted to be present and the resulting road-transport sound composite metric values at each raster cell.

We calculated separate composite metrics for each type of sound and for the day and night-times separately. We did this with geometric aggregation and equal weighting (Formula in SI (SI2)). In the future, alternative iterations of the metric with various weighting schemes could be tested and compared against relevant health outcome and/or well-being data when that data becomes available in Accra. A graphical illustration of how we created the aggregate metric combining sound levels and sound types can be seen in Fig. 6.

All analyses were performed in R (R version 3.6). The Random Forest models were implemented using the *randomForest* package in R and data visualizations with the *tmap*, *ggplot2*, and *ggdist* R packages.

## Electronic supplementary material

Below is the link to the electronic supplementary material.

Supplementary Material 1

## Data Availability

The predicted environmental sound level datasets (Lday, Lnight, Lden) which were reported on in a previous publication are hosted on Zenodo and can be downloaded from: https://zenodo.org/records/11223249 . The predicted sound type surface datasets derived from this work are available from the corresponding author on request.

## References

[1] ISO 12913-1. 2014 Acoustics — Soundscape Part 1: Definition and conceptual framework.

[2] Kang, J. et al. Sound environment: High-versus low-density cities. in *Designing high-density cities for socinvironmental sustainability* 163–180 (2010).

[3] Ratcliffe, E., Gatersleben, B. & Sowden, P. T. Bird sounds and their contributions to perceived attention restoration and stress recovery. *J. Environ. Psychol.* **36**, 221–228 (2013).

[4] Depledge, M. H., Stone, R. J. & Bird, W. J. Can natural and virtual environments be used to promote improved human health and wellbeing? *Environ. Sci. Technol.* **45**, 4660–4665 (2011). 21504154 10.1021/es103907m

[5] Basner, M. et al. Auditory and non-auditory effects of noise on health. *Lancet* **383**, 1325–1332 (2014). 24183105 10.1016/S0140-6736(13)61613-X PMC3988259

[6] World Health Organization. WHO environmental noise guidelines for the European region. (2018).

[7] Quartieri, J. et al. A review of traffic noise predictive noise models. *Recent Adv. Appl. Theor. Mech* 72–80 (2009).

[8] King, E. A. & Murphy, E. Environmental noise - ‘Forgotten’ or ‘ignored’ pollutant? *Appl. Acoust.* **112**, 211–215 (2016).

[9] Basner, M. & McGuire, S. WHO environmental noise guidelines for the European region: A systematic review on environmental noise and effects on sleep. *Int. J. Environ. Res. Public. Health*. **15**, 519 (2018). 29538344 10.3390/ijerph15030519 PMC5877064

[10] Guski, R., Schreckenberg, D. & Schuemer, R. WHO environmental noise guidelines for the European region: A systematic review on environmental noise and annoyance. *Int. J. Environ. Res. Public. Health*. **14**, 1539 (2017). 29292769 10.3390/ijerph14121539 PMC5750957

[11] Clark, C. & Paunovic, K. WHO environmental noise guidelines for the European region: A systematic review on environmental noise and cognition. *Int. J. Environ. Res. Public. Health*. **15**, 285 (2018). 29414890 10.3390/ijerph15020285 PMC5858354

[12] Stansfeld, S. A. & Matheson, M. P. Noise pollution: Non-auditory effects on health. *Br. Med. Bull.* **68**, 243–257 (2003). 14757721 10.1093/bmb/ldg033

[13] Kang, J. Urban soundscape. in Urban Sound Environment 43–107 (Routledge, (2017).

[14] Kazeem, Y. & Dahir, A. L. African cities are battling escalating noise pollution - but religion stands in the way. *Quartz Africa* (2018).

[15] Tong, H., Aletta, F., Mitchell, A., Oberman, T. & Kang, J. Increases in noise complaints during the COVID-19 lockdown in spring 2020: A case study in greater london, UK. *Sci. Total Environ.* **785**, 147213 (2021).

[16] Lee, P. J. & Jeong, J. H. Attitudes towards outdoor and neighbour noise during the COVID-19 lockdown: A case study in London. *Sustain. Cities Soc.* **67**, 102768 (2021). 33585168 10.1016/j.scs.2021.102768 PMC7866851

[17] Hong, A., Kim, B. & Widener, M. Noise and the city: leveraging crowdsourced big data to examine the spatio-temporal relationship between urban development and noise annoyance. *Environ. Plan. B Urban Anal. City Sci.* **47**, 1201–1218 (2019).

[18] Kaledzi, I. Ghana asks mosques to turn down the noise and use WhatsApp for call to prayer. (2018). https://www.dw.com/en/ghana-asks-mosques-to-turn-down-the-noise-and-use-whatsapp-for-call-to-prayer/a-43373007

[19] Samagwa, D., Mkoma, S. & Tungaraza, C. Investigation of noise pollution in restaurants in Morogoro municipality, tanzania, East Africa. *J Appl. Sci. Environ. Manag* **13**, (2010).

[20] Zakpala, R. N., Armah, F. A., Sackey, B. M. & Pabi, O. Night-time decibel hell: mapping noise exposure zones and individual annoyance ratings in an urban environment in Ghana. *Scientifica (Cairo)*. 1–11. 10.1155/2014/892105 (2014). 10.1155/2014/892105 PMC4127213 25136476

[21] Ebare, M. N., Omuemu, V. O. & Isah, E. C. Assessment of noise levels generated by music shops in an urban City in Nigeria. *Public. Health*. **125**, 660–664 (2011). 21875726 10.1016/j.puhe.2011.06.009

[22] Hoque, M. & Kabir, H. Traffic induced noise level in different places at the Dhaka capital City of Bangladesh. *Bangladesh J. Environ. Sci.* **38**, 41–46 (2020).

[23] Abankwa, E. & Agyemang, A. Impact of noise in the industry and commercial areas in ghana: case study of the Kumasi metropolis. *Int. J. Eng. Res. Appl.* **07**, 11–19 (2017).

[24] Knott, S. & Gyamfi, K. ‘If you complain they see you as evil’: accra’s religious noise problem. *The Guardian* (2019).

[25] van den Bosch, M. Ode sang. Urban natural environments as nature-based solutions for improved public health – A systematic review of reviews. *Environ. Res.* **158**, 373–384 (2017). 28686952 10.1016/j.envres.2017.05.040

[26] Ross, M. & Mason, G. J. The effects of preferred natural stimuli on humans’ affective states, physiological stress and mental health, and the potential implications for well-being in captive animals. *Neurosci. Biobehav Rev.* **83**, 46–62 (2017). 28916271 10.1016/j.neubiorev.2017.09.012

[27] Iyendo, T. O. Sound as a supportive design intervention for improving health care experience in the clinical ecosystem: A qualitative study. *Complement. Ther. Clin. Pract.* **29**, 58–96 (2017). 29122270 10.1016/j.ctcp.2017.08.004

[28] Medvedev, O., Shepherd, D. & Hautus, M. J. The restorative potential of soundscapes: A physiological investigation. *Appl. Acoust.* **96**, 20–26 (2015).

[29] Annerstedt, M. et al. Inducing physiological stress recovery with sounds of nature in a virtual reality forest - results from a pilot study. *Physiol. Behav.* **118**, 240–250 (2013). 23688947 10.1016/j.physbeh.2013.05.023

[30] Bakolis, I. et al. Urban mind: using smartphone technologies to investigate the impact of nature on mental well-being in real time. *Bioscience* **68**, 134–145 (2018). 29599549 10.1093/biosci/bix149 PMC5862247

[31] Ulrich, R. S. et al. Stress recovery during exposure to natural and urban environments. *J. Environ. Psychol.* **11**, 201–230 (1991).

[32] Radicchi, A. et al. Sound and the healthy City. *Cities Heal*. **5**, 1–13 (2020).

[33] Welsh Government. Noise and soundscape action plan. (2018).

[34] Mayor of London. The London Plan: The spatial development strategy for Greater London. (2021).

[35] City of London. Noise strategy 2016 to 2026. (2016).

[36] Mabin, A., Butcher, S., Bloch, R. & Peripheries Suburbanisms and change in sub-Saharan African cities. *Soc. Dyn.* **39**, 167–190 (2013).

[37] Wania, A., Kemper, T., Tiede, D. & Zeil, P. Mapping recent built-up area changes in the City of Harare with high resolution satellite imagery. *Appl. Geogr.* **46**, 35–44 (2014).

[38] Agyemang, F. S. K., Silva, E. & Poku-Boansi, M. Understanding the urban Spatial structure of Sub-Saharan African cities using the case of urban development patterns of a Ghanaian city-region. *Habitat Int.* **85**, 21–33 (2019).

[39] Addae, B. & Oppelt, N. Land-use/land-cover change analysis and urban growth modelling in the greater Accra metropolitan area (GAMA), Ghana. *Urban Sci.* **3**, 26 (2019).

[40] Angel, S., Parent, J., Civco, D. L., Blei, A. & Potere, D. The dimensions of global urban expansion: estimates and projections for all countries, 2000–2050. *Prog Plann.* **75**, 53–107 (2011).

[41] Sietchiping, R., Permezel, M. J. & Ngomsi, C. Transport and mobility in sub-Saharan African cities: an overview of practices, lessons and options for improvements. *Cities* **29**, 183–189 (2012).

[42] Imoro Musah, B., Peng, L. & Xu, Y. Urban congestion and pollution: A quest for cogent solutions for Accra City. *IOP Conf. Ser. Earth Environ. Sci* **435**, (2020).

[43] Ehebrecht, D., Heinrichs, D. & Lenz, B. Motorcycle-taxis in sub-Saharan africa: current knowledge, implications for the debate on informal transport and research needs. *J. Transp. Geogr.* **69**, 242–256 (2018).

[44] Amegah, A. K. & Agyei-Mensah, S. Urban air pollution in sub-Saharan africa: time for action. *Environ. Pollut*. **220**, 738–743 (2017). 27646170 10.1016/j.envpol.2016.09.042

[45] Clark, S. N. et al. Space-time characterization of community noise and sound sources in accra, Ghana. *Sci. Rep.* 1–12. 10.1038/s41598-021-90454-6 (2021). 10.1038/s41598-021-90454-6 PMC8160008 34045545

[46] Kang, J. Urban noise evaluation. in Urban Sound Environment (Routledge, (2017).

[47] Hammer, M. S., Swinburn, T. K. & Neitzel, R. L. Environmental noise pollution in the united states: developing an effective public health response. *Environ. Heal Perspect.* **122**, 115 (2014). 10.1289/ehp.1307272 PMC3915267 24311120

[48] European Environment Agency. *Environmental noise in Europe – 2020*. *European Environ. Agency* (2020).

[49] Chen, Y., Hansell, A. L., Clark, S. N. & Cai, Y. S. Environmental noise and health in low-middle-income-countries: A systematic review of epidemiological evidence. *Environ. Pollut*. **316**, 120605 (2023). 36347406 10.1016/j.envpol.2022.120605

[50] Salamon, J. & Bello, J. P. Deep convolutional neural networks and data augmentation for environmental sound classification. *IEEE Signal. Process. Lett.*10.1109/LSP.2017.2657381 (2017).

[51] Green, M. & Murphy, D. Environmental sound monitoring using machine learning on mobile devices. *Appl. Acoust.* **159**, 107041 (2020).

[52] OpenStreetMap. Planet dump. (2015). https://planet.openstreetmap.org

[53] Ghana Statistical Service. Ghana Population and Housing Census. (2010).

[54] Clark, S. N. et al. Spatial modelling and inequalities of environmental noise in accra, Ghana. *Environ. Res.* **214**, 113932 (2022). 35868576 10.1016/j.envres.2022.113932 PMC9441709

[55] Hill, A. P. et al. AudioMoth: evaluation of a smart open acoustic device for monitoring biodiversity and the environment. *Methods Ecol. Evol.* **9**, 1199–1211 (2018).

[56] Cartwright, M. et al. Sonyc urban sound tagging (Sonyc-Ust): a multilabel dataset from an urban acoustic sensor network. *Detect Classif. Acoust. Scenes Events* **2019** (2019).

[57] Fairbrass, A. J. et al. CityNet—Deep learning tools for urban ecoacoustic assessment. *Methods Ecol. Evol.*10.1111/2041-210X.13114 (2018).

[58] Salamon, J. & Bello, J. P. Deep convolutional neural networks and data augmentation for environmental sound classification. *IEEE Signal. Process. Lett.* **24**, 279–283 (2017).

[59] Changsong, Y., Barsim, K. S., Kong, Q. & Yang, B. Multi-level attention model for weakly supervised audio classification. *ArXiv Prepr arXiv* doi:180302353. (2018).

[60] Hong, K. Y., Pinheiro, P. O. & Weichenthal, S. Predicting outdoor ultrafine particle number concentrations, particle size, and noise using street-level images and audio data. *Environ. Int.* **144**, 106044 (2020). 32805577 10.1016/j.envint.2020.106044

[61] Arup & Cities Alliance. Future proofing cities: Ghana - metropolitan cities. (2016).

[62] Nathvani, R. et al. Measurement of urban vitality with time-lapsed street-view images and object-detection for scalable assessment of pedestrian-sidewalk dynamics (In review). *ISPRS J. Photogramm. Remote Sens.* 10.1016/j.isprsjprs.2025.01.038 PMC7617441 40027117

[63] Kang, J. Urban noise mitigation. In Urban Sound Environment 175–199 (Routledge, (2017).

[64] Kotzen, B. & English, C. *Environmental noise barriers: Guide to their visual and acoustic design*. (1999).

[65] Jia, Y., Ma, H. & Kang, J. Characteristics and evaluation of urban soundscapes worthy of preservation. *J. Environ. Manage.* **253**, 109722 (2020). 31666215 10.1016/j.jenvman.2019.109722

[66] Buxton, R. T., Pearson, A. L., Allou, C., Fristrup, K. & Wittemyer, G. A synthesis of health benefits of natural sounds and their distribution in National parks. *Proc. Natl. Acad. Sci. U S A*. **118**, 6–11 (2021). 10.1073/pnas.2013097118 PMC8040792 33753555

[67] Nathvani, R. et al. Beyond here and now: evaluating pollution Estimation across space and time from street view images with deep learning. *Sci. Total Environ.* **903**, 166168 (2023). 37586538 10.1016/j.scitotenv.2023.166168 PMC7615099

[68] Nathvani, R. et al. Characterisation of urban environment and activity across space and time using street images and deep learning in Accra. *Sci. Rep.* **12**, 20470 (2022). 36443345 10.1038/s41598-022-24474-1 PMC9703424

[69] Ghana Statistical Service. Greater Accra Population: Population by sex and district 2010 and 2019. (2019). https://statsghana.gov.gh/nationalaccount_macros.php?Stats=MTA1NTY1NjgxLjUwNg==/webstats/s679n2sn87

[70] Clark, S. N. et al. High-resolution Spatiotemporal measurement of air and environmental noise pollution in sub-Saharan African cities: pathways to equitable healthy cities study protocol for accra, Ghana. *BMJ Open.* 1–10. 10.1136/bmjopen-2019-035798 (2020). 10.1136/bmjopen-2019-035798 PMC7440835 32819940

[71] World Bank. 2014 Land Cover Classification of Accra, Ghana. (2014).

[72] U.S Geological Survey. Landsat products. https://www.usgs.gov/core-science-systems/nli/landsat

[73] Lopez Garcia, A. et al. A cloud-based framework for machine learning workloads and applications. *IEEE Access.* **8**, 18681–18692 (2020).

[74] ISO 12913-2. 2018 Acoustics - Soundscape - Part 2: Data collection and reporting requirements.

[75] Price, R. & Hallas, M. IN41A-02 Mapping every building and road in sub-Saharan Africa. in *AGU Fall Meeting* (2019).

[76] Barrington-Leigh, C. & Millard-Ball, A. The world’s user-generated road map is more than 80% complete. *PLoS One*. **12**, e0180698 (2017). 28797037 10.1371/journal.pone.0180698 PMC5552279

[77] Verdin, K. L. Digital Elevation Model (DEM) from the Hydrologic Derivatives for Modeling and Analysis (HDMA) database -- Africa. (2017). https://www.sciencebase.gov/catalog/item/591f6d02e4b0ac16dbdde1c7

[78] Agent, K. R. & Zegeer, C. V. *Propagation of Traffic Noise*. (1981).

[79] Ghinet, S. et al. Atmospheric propagation of aircraft acoustic signature from high altitude. *INTER-NOISE 2019 MADRID – 48th Int. Congr. Exhib. Noise Control Eng.* (2019).

[80] Corbisier, C. Living with noise. *U S Department Transportation* (2003). https://www.fhwa.dot.gov/publications/publicroads/03jul/06.cfm

[81] Aguilera, I. et al. Application of land use regression modelling to assess the Spatial distribution of road traffic noise in three European cities. *J. Expo Sci. Environ. Epidemiol.* **25**, 97–105 (2015). 25227731 10.1038/jes.2014.61

[82] Ragettli, M. S. et al. Statistical modeling of the Spatial variability of environmental noise levels in montreal, canada, using noise measurements and land use characteristics. *J. Expo Sci. Environ. Epidemiol.* **26**, 597–605 (2016). 26732373 10.1038/jes.2015.82

[83] Lee, M. et al. Land use regression modelling of air pollution in high density high rise cities: A case study in Hong Kong. *Sci Total Environ* **592**, (2017). 10.1016/j.scitotenv.2017.03.094 28319717

[84] Hoek, G. et al. A review of land-use regression models to assess Spatial variation of outdoor air pollution. *Atmos. Environ.* **42**, 7561–7578 (2008).

[85] Liu, Y. et al. Comparison of land use regression and random forests models on estimating noise levels in five Canadian cities. *Environ. Pollut*. **256**, 113367 (2020). 31662255 10.1016/j.envpol.2019.113367

[86] Harouvi, O., Ben-Elia, E., Factor, R., de Hoogh, K. & Kloog, I. Noise Estimation model development using high-resolution transportation and land use regression. *J. Expo Sci. Environ. Epidemiol.* **28**, 559–567 (2018). 29789670 10.1038/s41370-018-0035-z

[87] Wang, V. S. et al. Temporal and Spatial variations in road traffic noise for different frequency components in metropolitan taichung, Taiwan. *Environ. Pollut*. **219**, 174–181 (2016). 27814533 10.1016/j.envpol.2016.10.055

[88] Walker, E. D. et al. Spatial and Temporal determinants of A-weighted and frequency specific sound levels—An elastic net approach. *Environ. Res.* **159**, 491–499 (2017). 28865401 10.1016/j.envres.2017.08.034 PMC5903552

[89] Biau, G. & Scornet, E. A random forest guided tour. *Test* **25**, 197–227 (2016).

[90] Breiman, L. Random forests. *Mach. Learn.* **45**, 5–32 (2001).

[91] OECD. Handbook on constructing composite indicators: Methodology and user guide. (2008).

