# Supplementary material for: Moving beyond the noise: geospatial modelling of urban sound environments in a sub-Saharan African city
Source: Sci Rep. 2025 Jul 1;15:21403. doi: 10.1038/s41598-025-06537-1 (PMC12216697; doi:10.1038/s41598-025-06537-1)
Supplement: Supplementary file 1 — Supplementary Material 1 [file 41598_2025_6537_MOESM1_ESM.pdf]

Supplementary Information

Moving beyond the noise: geospatial modelling of urban sound environments in a sub-Saharan African city

Sierra N. Clark[1,2], Raphael E. Arku[3], Majid Ezzati[1,4,5], James Bennett[1,4], Ricky Nathvani[1,4], Abosede Sarah Alli[3], James Nimo[6], Josephine Bedford Moses[6], Solomon Baah[6], Allison Hughes[6], Samuel Agyei-Mensah[6], George Owusu[8], Mireille Toledano[1,4,9]* & Michael Brauer[10]*

*Joint senior authors

[1] Department of Epidemiology and Biostatistics, School of Public Health, Imperial College London, London, UK
[2] School of Health & Medical Sciences, City St George's, University of London, London, UK
[3] Department of Environmental Health Sciences, School of Public Health and Health Sciences, University of Massachusetts, Amherst, USA
[4] MRC Centre for Environment and Health, School of Public Health, Imperial College London, London, UK
[5] Regional Institute for Population Studies, University of Ghana, Accra, Ghana
[6] Department of Physics, University of Ghana, Accra, Ghana
[7] Department of Geography and Resource Development, University of Ghana, Accra, Ghana
[8] Institute of Statistical, Social & Economic Research, University of Ghana, Accra, Ghana
[9] Mohn Centre for Children's Health and Wellbeing, School of Public Health, Imperial College London, London, UK
[10] School of Population and Public Health, The University of British Columbia, Vancouver, Canada

Supplementary Information1. Predictor variables in the spatial Random Forest Models

Table SI1. Predictor variables considered for the spatial models

Variable type	Spatial calculation	Source (Date dataset created)
Land cover (*raster*)<br>*Industrial and business areas; informal residential; formal residential; other areas (e.g., forest, grassland, barren land, water)*	Mean area (m^2) within buffer	World Bank (2014)<br>20m x 20m [1]
Road-network (*Spatial line*)<br>*Major roads; secondary/ tertiary roads; minor roads; all roads*	Total length within buffer (m); distance to nearest (m)	OpenStreetMap (2019) [2]
Locations of places (*Spatial point*)<br>*Schools, hospitals, bus stations/ terminals, restaurants, bars and nightclubs, churches, mosques, shopping centres*	Presence/ absence within buffer; count within buffer	GooglePlaces (2020)
Normalized Difference Vegetation Index (*raster*)	Average NDVI value within buffer	United States Geological Survey (2020) – Landsat 8 imagery - 30m x 30m [3]
Population density within enumeration areas (*Spatial polygon*)	Average (pop/km^2) within buffer	Ghana census (2010) data [4]
Buildings footprints (*Spatial point*)	Count within buffer	Maxar/Ecopia.ai (2020)
Waterways (*Spatial line*)	Total length (m) within buffer	OpenStreetMap (2019) [5]
Elevation above sea level *(raster)*	--	U.S Geological Survey Digital Elevation Model (2017) (~90m) [6]

Supplementary Information 2: Composite Metric Formula

Formula A illustrates the structure of the composite metric for sound types and levels that we developed for the Greater Accra Metropolitan Area (GAMA): let a represent coordinate locations in the GAMA, and let q represent the individual components of the composite metric (i.e., sound levels and types). We calculated separate composite sound level-type metrics for each sound type and day and night-times.

$$IndicatorValue_a = \prod_{q=1}^{Q} x_{a,q} \quad \textit{for each of the five sound types and day and night}$$

Formula A. SoundType index

Supplementary Information 3: Distribution of detected sound types at 129 measurement sites

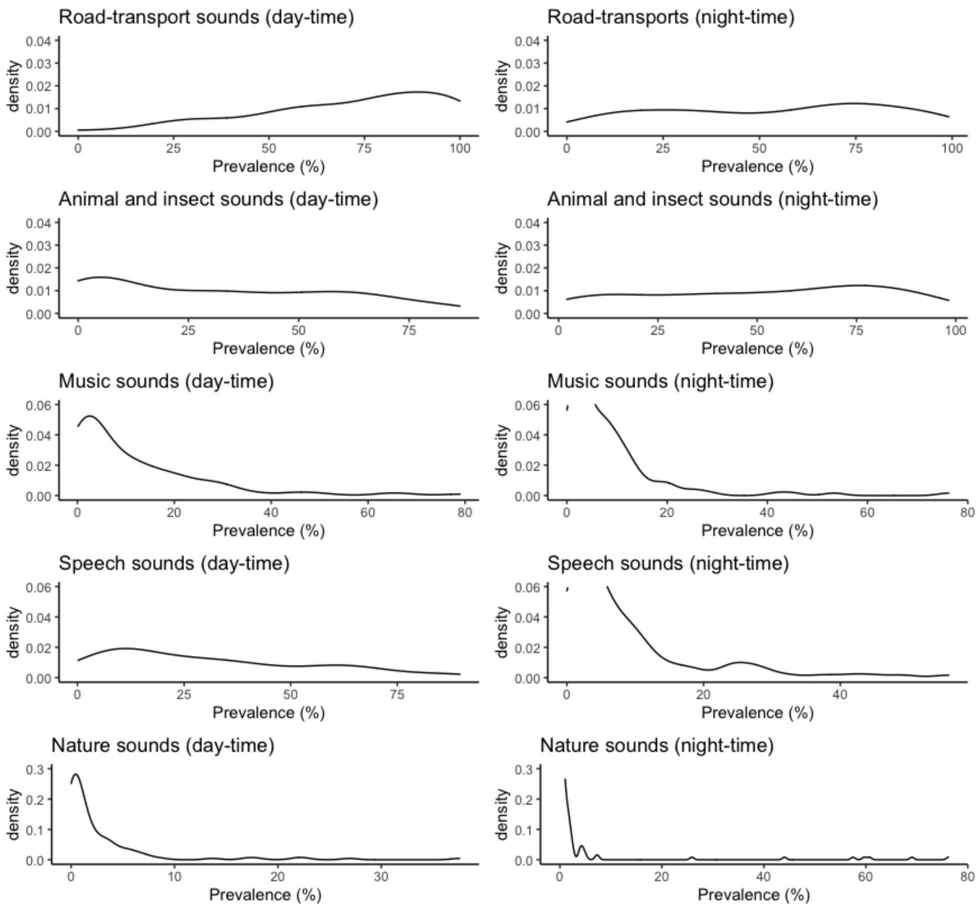

Figure SI1. Distributions (densities) of the percentage of time in the day and night that detected sound types were present (%) at each measurement site. Data represented in the figures are the raw acoustic classifications that were used to train the Random Forest land use regression prediction models.

Supplementary Information 4. Random Forest variable importance and out of sample model predictive accuracy

Table S2. The top three most important predictor variables within models based on reductions in model accuracy with random permutations.

	Day-time	Night-time
**Road-transport sounds models**	NDVI; Distance to nearest major road; Length of secondary/ tertiary roads	Length of Minor roads; Length of secondary/ tertiary roads; NDVI;
**Animal and insect sounds models**	NDVI; Length of secondary/ tertiary roads; Population density	Length of secondary/ tertiary roads; NDVI; Length of Major roads
**Music sounds models**	NDVI; Area of informal formal residential land use; Building density	Population density; NDVI; Building density
**Speech sounds models**	Building density; NDVI; Area of 'other' land use (e.g., forest/ grassland)	Population density; NDVI; Building density
**Nature sounds models**	NDVI; Building density; Area of informal formal residential land use	NDVI; Building density; Area of 'other' land use (e.g., forest/ grassland)

Table SI2. Model predictive accuracy with 10-fold cross validation holding 10% of random sites.

	**10-fold cross validation with 10% random sites**				
**Dependant variable: % of time sound type is present [Range: 0-100%]**	Absolute median error	Absolute mean error	Mean error (ME)	Correlation (r)	r^2
**Day-time models**					
**Road-transport**	10.04%	13.65%	0.65%	0.69	0.48
**Animal and insect**	11.13%	14.39%	-0.56%	0.72	0.52
**Music**	6.84%	9.26%	-0.67%	0.42	0.18
**Speech**	13.57%	15.65%	-0.30	0.60	0.36
**Night-time models**					
**Road-transport**	15.79%	17.91%	1.11%	0.68	0.46
**Animal and insect**	16.57%	19.16%	-0.59%	0.59	0.34
**Music**	4.16%	6.23%	-0.53%	0.38	0.14
**Speech**	3.83%	6.05%	0.46%	0.53	0.28

10-fold cross validation of 10% random sites: Holding out a random sample of data from 10% of measurement sites, 10-times (sampling without replacement), as the testing dataset, while training on the data from the other 90% of sites. Model accuracy results for nature sounds is in SI5.

The mean error (ME), which is a measure of bias, was near zero among rotating sites, indicating no systematic under or over prediction on average. We did not find evidence of residual spatial autocorrelation in model residuals and the Moran's I statistic of spatial autocorrelation indicated a tendency towards spatial randomness of the residuals (Moran's I statistic: -0.04 to 0.07).

Supplementary Information 5: Nature sound model predictive accuracy

Table SI3. Nature sounds model out of sample predictive accuracy.

	10-fold cross validation with 10% random sites			
**% of time nature sounds present [Range: 0-100%]**	Absolute median error	Absolute mean error	Mean error (ME)	Correlation
**Nature (day-time)**	1.64%	2.96%	-0.10%	0.18
**Nature (nigh-time)**	1.83%	6.07%	-0.17%	0.01

Supplementary Information 6. Predicted presence (%) of road-transport and animal and insect sounds near road-networks

Table SI4. The average percentage of time (%) sounds were predicted as present near major roads, secondary/tertiary roads, and minor roads in the Greater Accra Metropolitan Area. Data are expressed as medians and interquartile ranges (IQR).

	**Day-time models**		**Night-time models**	
	Road-transport sounds (%)	Animal and insect sounds (%)	Road-transport sounds (%)	Animal and insect sounds (%)
**Near major roads**	80 (74, 85)	35 (23, 46)	62 (52, 71)	43 (34, 52)
**Near secondary and/or tertiary roads**	71 (56, 81)	41 (25, 54)	51 (39, 63)	50 (40, 76)
**Near minor roads**	56 (46, 69)	55 (43, 62)	34 (27, 46)	67 (56, 74)

Nearness to a road type was defined as the site location was within 100m buffer of the road.

Supplementary Information 7. Distributions of nighttime predicted sound type prevalence (% of time present) across areas with varying predicted sound levels (Lnight, dBA)

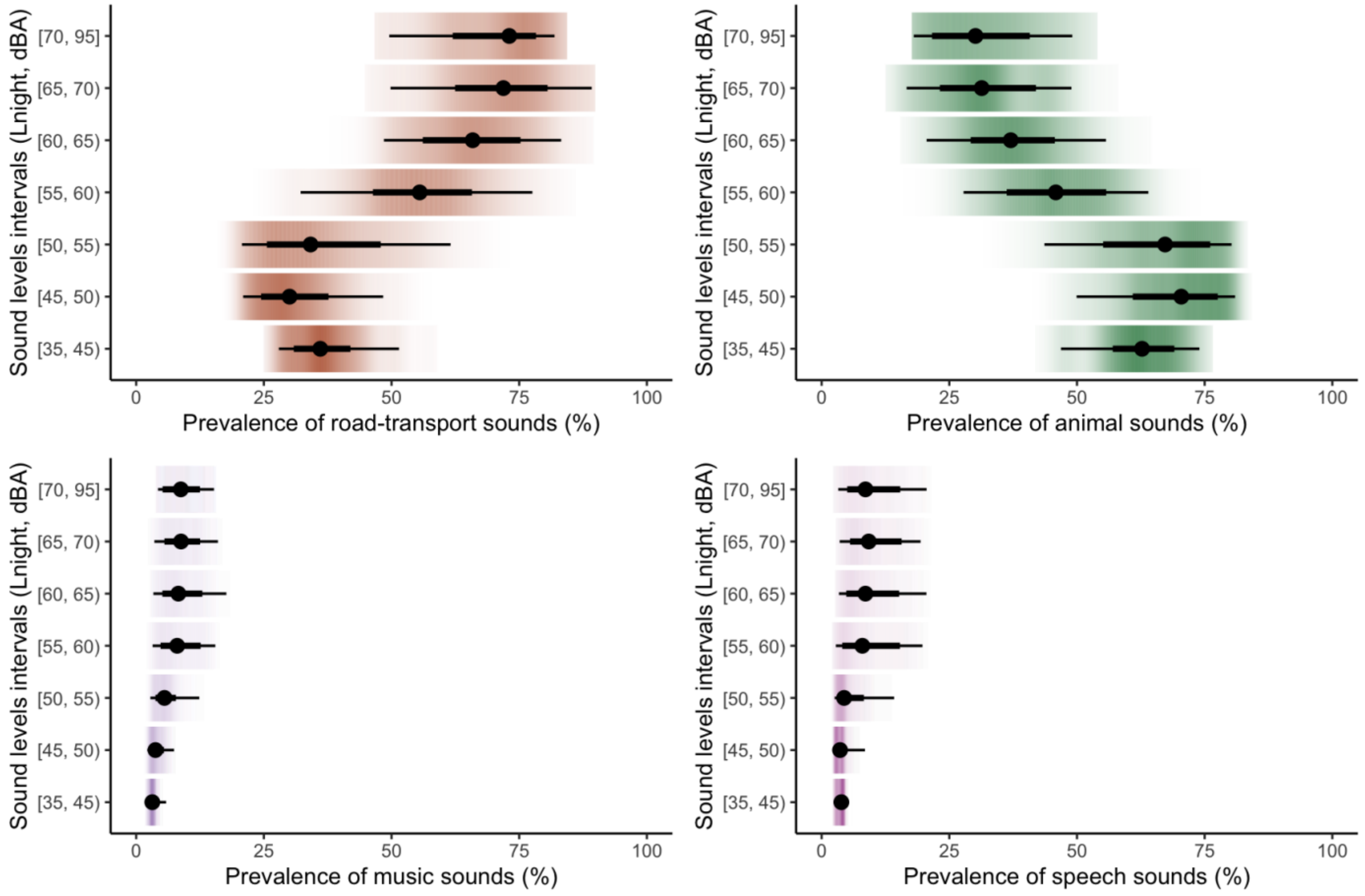

Figure SI2. Distributions of nighttime predicted sound type prevalence (% of time present) across areas with varying predicted sound levels (Lnight, dBA). Circular points represent the sample median, black horizontal lines the quartile range, and the coloured regions the density of the sample distribution.

References

1. World Bank. *2014 Land Cover Classification Of Accra, Ghana*. (2014).
2. Barrington-Leigh, C. & Millard-Ball, A. The world's user-generated road map is more than 80% complete. *PLoS One* **12**, e0180698 (2017).
3. U.S Geological Survey. Landsat products. https://www.usgs.gov/core-science-systems/nli/landsat.
4. Ghana Statisical Service. Ghana Population and Housing Census. (2010).
5. OpenStreetMap. Planet dump. https://planet.openstreetmap.org (2015).
6. Verdin, K. L. Digital Elevation Model (DEM) from the Hydrologic Derivatives for Modeling and Analysis (HDMA) database -- Africa. *2017* https://www.sciencebase.gov/catalog/item/591f6d02e4b0ac16dbdde1c7.
